# Totally Laparoscopic Pylorus-Preserving Gastrectomy (TLPPG) is Safe and Effective for Early Gastric Cancer Treatment

**DOI:** 10.14789/jmj.JMJ23-0018-OA

**Published:** 2023-11-16

**Authors:** GUO WEI, HE ZHIPENG, SU SHI, CAROLINE NADIA FEDOR, MEI XIANGHUANG, WANG YANGYANG, ZHANG KE, GUAN XIAOQI, BROCK MALCOLM V., HAJIME ORITA, TETSU FUKUNAGA

**Affiliations:** 1 Dept of GI Surgery, Changzhi Medical College, Affiliated Heji Hospital, Shanxi, China; 1 Dept of GI Surgery, Changzhi Medical College, Affiliated Heji Hospital, Shanxi, China; 2 Graduate School of Changzhi Medical College, Shanxi, China; 2 Graduate School of Changzhi Medical College, Shanxi, China; 3 Department of Gastroenterological Surgery (Upper), Juntendo University School of Medicine, Tokyo, Japan; 3 Department of Gastroenterological Surgery (Upper), Juntendo University School of Medicine, Tokyo, Japan; 4 Johns Hopkins University School of Medicine, Department of Surgery, MD, USA; 4 Johns Hopkins University School of Medicine, Department of Surgery, MD, USA; 5 Juntendo University International Collaboration Research Institute, Tokyo, Japan; 5 Juntendo University International Collaboration Research Institute, Tokyo, Japan

**Keywords:** laparoscopic pylorus-preserving gastrectomy, post-gastrectomy syndrome, post-gastrectomy syndrome assessment scale-37, early gastric cancer

## Abstract

**Background:**

Compared to distal gastrectomy (DG), pylorus-preserving gastrectomy (PPG), a peristaltic function-preserving surgery for early gastric cancer (EGC), is advantageous as it leads to a more improved nutritional status and quality of life (QOL) of patients. In recent years, total laparoscopic PPG (TLPPG), an anastomosis which is performed intracorporeally, has increasingly replaced laparoscopic-assisted PPG (LAPPG) due to its minimal invasiveness.

**Aim:**

To evaluate the safety and feasibility of TLPPG in terms of perioperative efficacy.

**Patients:**

Three patients underwent TLPPG in the Affiliated Hospital of Changzhi Medical College from September 2021 to March 2022.

**Methods:**

Surgical safety analysis: Our three cases (TLPPG group) were compared to data from the CLASS-02 study, which collected data from multiple centers across China for the laparoscopic total gastrectomy (LTG group). The CLASS-02 study provides data from the most invasive type of gastric surgery, providing solid comparative data to our own.

Postoperative short-term efficacy analysis: Patient questionnaire responses provided data on postoperative nutritional and QOL status. Results from our three cases were compared to the Japanese multicenter data PGSAS-37 (PGSAS group).

**Results:**

There were no complications or deaths occurred during or after operation in our cases. Compared to the PGSAS group, our cases scored lower for abdominal pain, dyspepsia, and weight loss.

**Conclusion:**

Although more case information is needed, our findings demonstrate that TLPPG may be a possible and effective treatment for EGC in China, similar to that in Japan.

## Introduction

Early gastric cancer (EGC) has been able to achieve extremely high cure rates through the use of minimally invasive (MI) and function‐preserving (FP) surgeries in east Asian countries^1)^. Most notably, pylorus-preserving gastrectomy (PPG) aims to prevent dumping syndrome and maintains the nutritional status of patients being treated for EGC located in the middle third of the stomach^1, 2)^. Specifically, laparoscopic assisted PPG (LAPPG) has been used in recent years to perform anastomosis intracorporeally. With current advances in technology, LAPPG has gradually been replaced by total laparoscopic PPG (TLPPG), a procedure that produces more cosmetically desirable results, less patient pain and risk of infection, and better postoperative quality of life (QOL). TLPPG has become the standard in Japan and Korea^3, 4)^.

However, unlike Japan and Korea, China has not yet adopted TLPPG, perhaps due to the low EGC diagnosis rate and the technical complexity of the procedure. None-the-less, China still has a number of gastric cancer diagnoses every year that require optimal treatment. In 2022 alone, about 500,000 new cases presented^5)^, of which EGC was as great as 20%^6)^.

In order to initiate the use of TLPPG in China, it is necessary to ensure that newly learned TLPPG procedures are done properly and can yield similar results to procedures done regularly. Therefore, we performed TLPPG on three Chinese patients (TLPPG group) and quantitatively observed their postoperative conditions and QOL status to see if we could produce similar results to the CLASS-02 study^7)^(LTG group) in China^8)^ the multicenter data PGSAS-37 (PGSAS group) in Japan.

## Materials and methods

### Patients

Three EGC patients underwent TLPPG at the Affiliated Heji Hospital of Changzhi Medical College from September 2021 to March 2022. All patients completed the PGSAS-37 questionnaire after the operation. The clinical, perioperative, pathological, and PGSAS-37 questionnaire data were retrospectively analyzed. Clinical data included time after surgery, age, gender, preoperative body mass index (BMI), pathological stage, surgical approach, extent of lymph node dissection, and combined resection. The gastric tumors were pathologically staged according to Japanese guidelines of gastric cancer treatment. The study protocol was approved by the Ethics Committee of the Affiliated Heji Hospital of Changzhi Medical College (Approval No.202005). The need for informed consent was waived in view of the retrospective and observational nature of the study.

### Surgical procedure

**Figure 1:**
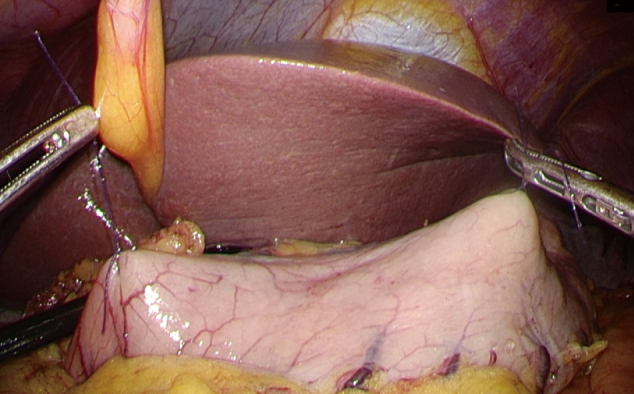
The upper and lower margins of the tumor were located by endoscopy and laparoscopy during the operation, and marked with suture.

**Figure 2:**
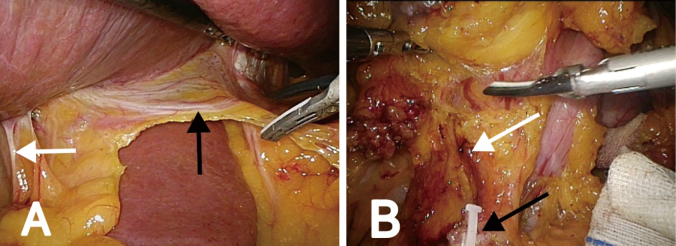
Preservation of the vagus nerve and inferior pyloric vessels. (A) The hepatic branch of vagus nerve (black arrowhead) is located at the omentum near the liver and the pyloric branch (black arrowhead) in the middle of the hepatoduodenal ligament. (B) The severed right gastroepiploic vessels (black arrowhead) at the beginning of the vessels and the preserved inferior pyloric vessels (white arrowhead).

**Figure 3:**
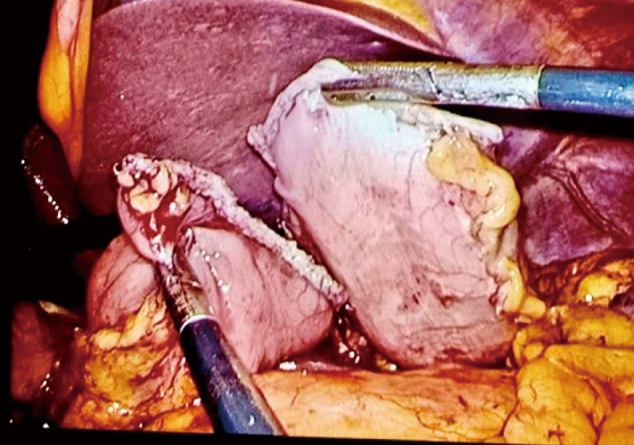
Straight-line cutting closers were used to remove the middle part of the stomach from the upper and lower edges at the marked site (A). The specimen removed through assisted small incision is shown in (B).

**Figure 4:**
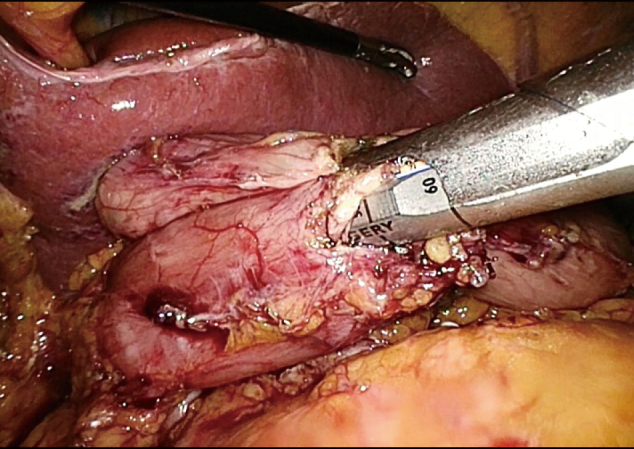
Anastomosis was performed by an endoscopic linear stapler.

**Figure 5:**
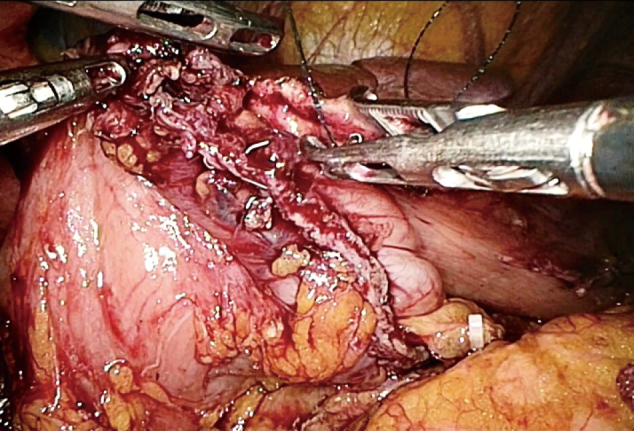
The anterior wall of the proximal and distal residual stomach was sutured and the common opening was closed.

Our TLPPG operation focused on the upper and lower incisional margins, the protection of nerves and blood vessels, and the resection and reconstruction of the stomach, which we elaborate in the following three parts:

1. At the beginning of the operation, the tumor was accurately located using laparoscopy and endoscopy. The upper and lower boundaries that were identified before operation, were marked with sutures (Figure 1).

2. The hepatic branch of the vagus nerve and the pyloric branch needed to be preserved during the operation. First, the assistant held up the visceral surface of the liver, exposed the lesser omentum, and cut off the right gastric artery. The second branch of the right gastric artery was protected to preserve the blood supply to the lesser curvature of the antrum and the innervation of the pyloric branch of the vagus nerve. We cut the omentum along the right gastric vessel, hepatoduodenal ligament, and hepatic branch of the vagus nerve, and then cut off the anterior gastric branch at the distal end of the hepatic branch of the vagus nerve. In order to avoid thermal injury, the distance between the head of the ultrasonic scalpel and the vagus nerve was more than 5 mm (Figure 2A). The inferior pyloric vessels also need to be protected to preserve the blood supply to the pylorus. Along the fusion fascia, the pancreatic head, duodenal bulb, and right gastroepiploic vessels were separated and exposed layer by layer, and the No.6v and No.6a lymph nodes were cleared near the pancreas. Then, the gatrodoudenalteria was dissected by tracing the right gastroepiploic artery and the inferior pyloric artery along the GDA to identify positional relationships. Following this, the right gastroepiploic vessel was cut, and the inferior pyloric artery was retained (Figure 2B).

3. The middle part of the stomach was removed with two straight-line cutting closers from the upper and lower boundaries at the marked site, thus preserving the pylorus and cardia. To reduce the risk of postoperative gastric emptying disorder, a sufficient antrum length was maintained. If conditions permitted, more than 3 cm of the antrum was kept, and the specimen was transected more than 2 cm from the distal edge of the tumor. After resection, the distal and proximal ends of the stomach were obtained (Figure 3). The lateral stapler was used to anastomose the posterior wall of the proximal stomach with the anterior wall of the distal stomach (Figure 4). A 3-0 barbed thread was used to suture the common opening to achieve full-thickness suture (Figure 5).

### CLASS-02 study

The Chinese Laparoscopic Gastrointestinal Surgery Study (CLASS-02) was a prospective, multicenter, randomized clinical trial that compared the safety of laparoscopic total gastrectomy (LTG) versus open total gastrectomy (OTG) with D1+/D2 lymphadenectomy for patients with clinical stage I (T1N0M0, T1N1M0, T2N0M0) gastric cancer in the upper or middle third of the stomach. From January 2017 to September 2018, a total of 227 patients were enrolled. The primary outcome was the morbidity and mortality within 30 days following surgery. The secondary outcomes were the recovery courses and the postoperative hospital stays. We used the LTG group data of the CLASS-02 trial as the control group and compared perioperative data from the TLPPG and LTG groups.

### PGS & QOL assessment

The PGSAS-37^9, 10)^ questionnaire consists of 37 questions, of which 15 are from the Gastrointestinal Symptom Assessment Scale and 22 are clinically relevant questions that have been proposed by the Japanese Postgastrectomy Syndrome Working Party (JPGSWP) (supplemental Table 1) (https://www.jsgp.jp/index.php?page=about_pgsas). These questions are summarized into nine subscales with a total of 17 primary outcomes, including assessment of esophageal reflux, abdominal pain, diet-related discomfort, dyspepsia, diarrhea, constipation, dumping syndrome, food quality, and dissatisfaction with daily life. The main findings consist of three categories: symptoms, life status, and QOL (supplemental Table 2). Higher scores for food intake per meal, daily food intake, appetite, hunger, satiety, food quality, and body weight change indicated better results, while lower scores for all other aspects indicated better results. The questionnaires were distributed by the physician at the time of the follow-up.

The PGSAS-37 questionnaire was completed by the three TLPPG patients in our center and by 313 PPG patients from the PGSAS group. The two groups were compared in terms of PGS symptoms and QOL scores.

## Results

### Three cases performed successfully based on perioperative status.

We counted and described the preoperative and postoperative data of three patients, including 2 males and 1 female. The average age was 60 ± 3.6 years old, and the average BMI was 24.3 ± 1.8. The clinical stage was T1. The operation was completed under total laparoscopy, and the lymph node dissection was D1+. We followed up 3 patients after operation and filled in the PGSAS-37 questionnaire. Table 1 is the original data of 17 subscales, which provides evidence for our data comparison.

**Table 1:** Original data of 17 subscales

		Case 1	Case 2	Case 3	Mean	SD
Symptom	Esophageal reflux scale	2.25	1.75	1.89	1.88	0.21
	**Abdominal pain scale**	1.25	1.67	1.33	1.42	0.18
	Diet related discomfort scale	2.33	1.67	2.67	2.22	0.42
	**Dyspepsia scale**	1.67	1.75	2.00	1.81	0.14
	Diarrhea scale	2.33	1.00	2.00	1.78	0.57
	Constipation scale	1.33	2.33	2.33	2.00	0.47
	Dumping syndrome scale	2.33	2.00	1.00	1.78	0.57
	Overall symptom score	1.93	1.74	1.89	1.85	0.08
Living status	**Body weight change rate (%)**	-3.6%	-7.1%	-3.2%	-4.7%	1.8%
	Amount of food eaten at a time	8.00	5.00	6.00	6.33	1.25
	The need for extra meals	1.00	2.00	1.00	1.33	0.47
	Food intake quality scale	3.00	4.67	4.67	4.11	0.79
	service ability	1.00	4.00	2.00	2.33	1.25
QOL	**Symptom dissatisfaction**	2.00	1.00	1.00	1.33	0.47
	Dietary dissatisfaction	4.00	1.00	3.00	2.67	1.25
	Job dissatisfaction	2.00	1.00	2.00	1.67	0.47
	Dissatisfaction with daily life subscale	2.67	1.00	2.00	1.89	0.68

The PPG group in China compared the safety of LTG and OTG for EGC using the perioperative data of 214 patients (105 cases for LTG and 109 cases for OTG). In order to demonstrate the short-term safety of TLPPG, we utilized the LTG arm of CLASS-02 trial as the control group and compared the perioperative data of the TLPPG and LTG groups. As shown in Table 2, the number of retrieved lymph nodes in the TLPPG group (17 ± 2.2) was less than that in the LTG group (35 ± 12.7). The surgery time, estimated blood loss, time to first flatus, etc. were similar between the TLPPG and LTG groups. Furthermore, no complications or deaths occurred among our patients during or after operation (Table 3). The overall complication and mortality rates were similar between the two groups. In terms of intraoperative and postoperative complications, including surgery-related and system-related complications, no difference was noted between the two groups. These results indicated that TLPPG is a safe option.

**Table 2:** Surgical results & outcome

	TLPPG group (*n* = 3)	LTG group (*n* = 105)
Surgical time (min)	193 ± 12	230 ± 67.3
Estimated blood loss (ml)	60 ± 8	92 ± 109.6
Time to first flatus (d)	3.3 ± 0.5	3.1 ± 0.9
Open conversion	0（0%）	2 (1.9%)
No. of retrieved lymph nodes	17 ± 2.2	35 ± 12.7
Time to ambulation (d)	24.3 ± 1.2	40.6 ± 20.6
Postoperative hospital stay	10 ± 0.8	10.9 ± 5.1

**Table 3:** Morbidity and mortality

Morbidity type/mortality	TLPPG group (*n* = 3)		LTG group (*n* = 105)	
	No.	Rate, % (95% CI)	No.	Rate, % (95% CI)
All complications	0	0	24	22.9(0.15〜0.3)
Mortality	0	0	1	1.0 (0〜2.8)

### The PGS and QOL are similar between PGSAS and TLPPG groups.

The clinicopathological features of the patients were summarized in Table 4. Because TLPPG is a less invasive procedure, the observation period after surgery was shorter than that in the PGSAS group (6.7 ± 4.1 months vs 38.4 ± 27.7 months). The preservation of the celiac branch of vagal nerve and combined organ resection in TLPPG group were less than the PGSAS group. The average age of the TLPPG group was 60.0 ± 3.6 years and that of the PGSAS group was 61.5 ± 8.7 years. There were no differences between the two groups in terms of gender, preoperative body mass index, surgical method, and lymph node dissection method.

**Table 4:** Clinical and pathological characteristics results

	TLPPG group (*n* = 3)	PGSAS group (*n* = 313)
Postoperative time (month)	6.7 ± 4.1	38.4 ± 27.7
Age	60 ± 3.6	61.5 ± 8.7
Gender		
Male	2	183
Female	1	126
Preoperative BMI	24.3 ± 1.8	22.7 ± 3.0
Stages		
Ⅰ	3	313
Ⅱ	0	0
Ⅲ	0	0
Ⅳ	0	0
Surgical approach		
peritoneoscope	3	136
open abdomen	0	173
Degree of lymph node dissection		
D1+	3	252
D1	0	6
D2	0	8
The celiac branch reservation (absence/presence)		
Absence	1	213
Presence	2	87
Combined resection (absence/presence)		
absence	1	12
presence	2	279

The PGSAS-37 scores for 17 symptoms were summarized in Table 5. The abdominal pain (1.42 ± 0.18 vs 1.64 ± 0.73) and dyspepsia (2.83 ± 0.47 vs 2.01 ± 0.88) scores were higher in the TLPPG group compared to the PGSAS group. Furthermore, the percentage body weight loss was lower in the TLPPG group than in the PGSAS group (4.7% vs 6.9%). There were no differences in the amount of food taken per meal, the need for additional meals, and the quality of intake between the TLPPG and PGSAS groups. Finally, the scores of QOL subscales, including dissatisfaction with symptoms, diet, work, and daily life, were also similar in both groups.

**Table 5:** PGSAS-37 main symptom scores

		TLPPG group (*n* = 3)		PGSAS group (*n* = 313)	
		Mean	SD	Mean	SD
Symptom	Esophageal reflux scale	1.88	0.21	1.70	0.82
	**Abdominal pain scale**	1.42	0.18	1.64	0.73
	Diet related discomfort scale	2.22	0.42	2.11	0.87
	**Dyspepsia scale**	1.81	0.14	2.01	0.88
	Diarrhea scale	1.78	0.57	1.84	0.97
	Constipation scale	2.00	0.47	2.24	1.08
	Dumping syndrome scale	1.78	0.57	1.75	0.94
	Overall symptom score	2.00	0.14	1.89	0.67
Living status	**Body weight change rate (%)**	-4.7%	1.8%	-6.9%	7.0%
	Amount of food eaten at a time	6.33	1.25	7.02	1.87
	The need for extra meals	1.33	0.47	1.75	0.75
	Food intake quality scale	4.11	0.79	3.76	0.93
	Service ability	2.33	1.25	1.77	0.95
QOL	**Symptom dissatisfaction**	1.33	0.47	1.80	0.94
	Dietary dissatisfaction	2.67	1.25	2.23	1.11
	Job dissatisfaction	1.67	0.47	1.67	0.91
	Dissatisfaction with daily life subscale	1.89	0.68	1.90	0.83

### The self-nutrition indices also performed well.

We also compared the pre-operative and post-operative nutritional status of our three patients. As shown in Table 6, the BMI, albumin (ALB), and hemoglobin (HGB) level three months after operation were similar to the respective pre-operative values.

**Table 6:** Self-nutrition indices results

	Preoperative	3 months after surgery
BMI	24.3 ± 1.8	23.3 ± 1.1
ALB（g/L）	44.4 ± 2.8	45.4 ± 2.1
HGB	144.3 ± 9.0	142.3 ± 9.2

## Discussion

Intracorporeal and robotic surgery for resections and anastomosis has become popular in GI surgery around the world due to recent technological advancements^11)^. Operation with good EGC prognosis requires highly skilled MI and FP methods. However, full total operations have been increasing in Asia.

Total laparoscopic PPG (TLPPG) is one of many typical examples. This procedure prevents dumping syndrome, maintains the nutritional status, and requires a cosmetically small incision which is less painful and has a quicker recover time^3)^. Because of these benefits, we introduced TLPPG, a technique not extensively used in China, to our clinic with the anticipation of increasing EGC diagnoses and the aim of offering better postoperative outcomes to Chinese patients with this disease.

To successfully perform the procedure and obtain ideal results, there are two major challenges that physicians must overcome. First, the operator needs to develop a scientific system for diagnosis and treatment of gastric cancer, such as preoperative positioning, intraoperative positioning, frozen section analysis of the cutting edge in the perioperative. Second, the operator needs to protect the hepatic branch and pyloric branch of the vagus nerve, as well as protect the blood vessels under the pylorus, and clean up the No.6a and No.6v lymph nodes.

Furthermore, it is worth emphasizing that: 1. A scientific diagnosis and treatment system for gastric cancer should be developed in conjunction with relevant departments, such as the Endoscopy Department, Pathology Department and other relevant disciplines. 2. The operator should be familiar with the vagus nerve and its branches. The distance between the ultrasonic scalpel head and the vagus nerve should be more than 5 mm to avoid thermal injury. 3. The operator should dissect along GDA to reveal the position relationship between the right gastroepiploic artery and the inferior pyloric artery, and then disconnect and retain these vessels.

To estimate the quality of our three TLPPG cases, we were required to measure the MI and FP. We used the CLASS-02 study (LTG) in China to estimate MI and evaluate surgical safety because LTG is the most difficult full total operation in China for EGC. There were no differences regarding blood loss, surgical time, hospital stay, and the incidence of complications compared with LTG in China. These results indicated that our initial TLPPG procedures were a safe option in our center. As for FP, we used PGSAS-37, the Japanese standard post operative score, which discusses post gastrectomy status.

Compared with other indicators, the postoperative efficacy of patients is difficult to evaluate since many symptoms cannot be quantified. In order to better quantify the postoperative state of our patients, we assessed the PGS score and QOL using the PGSAS-37 questionnaire by the Japanese national database that has been designed to evaluate functional parameters after gastrectomy. When comparing postoperative short-term efficacy, most symptom subscales and overall symptom scores were similar in both groups, with the most similar results between postoperative QOL scores. The abdominal pain, dyspepsia, and body weight loss in the PPG group yielded better results than that of the PGSAS group. Data from the PGSAS group were obtained from open and laparoscopic surgeries in multiple centers across Japan beginning in 2015.

In our center, we adopted the latest TLPPG technique, allowing us to obtain desirable patient outcomes for EGC. Abdominal pain was reduced by TLPPG since it was less invasive (Table 4), it's mainly manifested in stomach ache. The smaller incision led to less tissue damage compared to conventional laparotomy and laparoscopic-assisted surgery^12)^, this will reduce the obstruction caused by adhesion of the stomach or intestines and reduce abdominal pains. As compared to LAPPG, the small incisions required for TLPPG remained uniform in size between patients and were independent of patient factors, leading to a potential advantage for using the technique^13-15)^. The score of the dyspepsia subscale was also lower than that of the PGSAS group. Studies have shown that, delayed gastric emptying (DGE)^16, 17)^ and decreased receptive relaxation function^18)^ can cause dyspepsia. DGE is the most common and prominent complication after PPG, which may be related to factors such as blood supply to the gastric antrum^19)^ and the retention length of gastric antrum^20)^. According to Fukunaga^19-21)^, preserving the infrapyloric vessel and the first branch of the right gastric vessel can greatly reduce DGE. The initial cases of PPG involved maintaining the length of the gastric antrum at 1.5 cm. With this antral length, the incidence of postoperative DGE ranged from 23% to 40%^19-21)^. Multiple retrospective studies have shown that in order to exert the functions of the preserved gastric antrum and pylorus and to reduce DGE, the reserved length of the gastric antrum above the pyloric canal should be at least 2.5-3 cm^2, 22-24)^. Some centers even require the preserved length of gastric antrum to be more than 4 cm^25, 26)^.

During operation in our center, we performed nerve protection and preserved a sufficient length of gastric antrum (3-4 cm) as well as the blood vessel under the pylorus and the first branch of the right gastric vessel. Gastrointestinal contrast examination during the perioperative period and three months post-surgery showed patency, and the incidence of DGE was 0. Sufficient antrum preservation ensured a large residual gastric cavity, maximal preservation of the receptive relaxation function of the stomach, and minimal risk of dyspepsia. In addition, the rate of body weight loss was also better than the PGSAS group, primarily due to the reduction in DGE and dyspepsia, along with good nutrition absorption without obvious diet-related discomfort.

Despite observing the benefits of TLPPG in our cases, it is important to note that a weakness of this study was the extremely low number of our cases in comparison to that of previously collected data in the CLASS-02 and PGSAS-37 studies.

## Conclusion

Having learned TLPPG from Professor Fukunaga, our clinic has been introduced to this innovative surgical technique and has observed its advantages. Although this method was only tested on three patients in our clinic thus far, our cases produced similar postoperative outcomes to those in Japan, suggesting that if done in larger number, more robust conclusions may be made. Never-the-less, our findings provide beginning evidence that this technique will be safe and effective for use in clinics across China.

## Funding

No funding was received.

## Author contributions

GW, HZP and HO designed the study; GW, HZP, MXH and WYY treated patients and collected material and clinical data from the patients; HZP, ZK and GXQ analyzed data; GW wrote the paper; All authors read and approved the final manuscript. MV and CF, native speaker, corrected English.

## Conflicts of interest statement

The authors declare that there are no conflicts of interest.

**Supplemental Table 1:** PGSAS-37 evaluation items

	Item	Subscales
Symptom	1 Abdominal pain*	Esophageal reflux sub-scale (Items 2, 3, 5 and 16)
	2 Heartburn	Abdominal pain sub-scale (Items 1, 4 and 20)
	3 Trans-acid	Sub-scale of diet-related irritability (Items 17-19)
	4 Fasting stomachache	Sub-scale for dyspepsia (Items 6-9)
	5 Nausea and vomiting	Diarrhea sub-scale (Items 11, 12 and 14)
	6 Borborygmus	Constipation sub-scale (Items 10, 13 and 15)
	7 Stomach distension	Dumping syndrome subscale (Items 22, 23 and 25)
	8 hiccups	
	9 Increased flatus	Total symptom score (over 7 subscales)
	10 Constipation	
	11 Diarrhea	
	12 Soft stool	
	13 Hard stool	
	14 Urgent need to defecate	
	15 Incomplete defecation	
	16 Bile reflux	
	17 Dysphagia	
	18 Postprandial stagflation	
	19 Early satiety	
	20 Lower abdominal pain	
	21 Number and type of early dumping syndrome	
	22 Early dumping, general symptoms	
	23 Early dumping, abdominal symptoms	
	24 Number and type of late dumping syndrome	
	25 Advanced dumping syndrome	
Living status	26 Amount of food consumed per meal	
	27 Daily food intake	
	28 Staple food frequency	
	29 Supplemental frequency	Intake quality subscale (Items 30-32)
	30 Appetite	
	31 Starvation	
	32 Feeling of satiety	
	33 The need for a meal	
	34 Working capacity	
QOL	35 Symptom dissatisfaction	
	36 Dissatisfaction with diet	Dissatisfaction with life sub-scale (Items 35-37)
	37 Dissatisfaction with one's work	

*: Abdominal pain mainly refers to stomach ache

**Supplemental Table 2:** Main results in the three categories

Category	Key findings
Symptoms	Esophageal reflux scale
	Abdominal pain scale
	Sub-scale of diet-related discomfort
	Dyspepsia sub-scale
	Diarrhea subset scale
	Constipation subscale
	Dumping syndrome subscale
	Total symptom score
Living conditions	
Weight	Weight change (%)
	
Diet (quantity)	Amount of food taken in per meal (%)
	The need for extra food
Diet (quality)	Intake quality sub-scale
Work	Service ability
QOL	Dissatisfaction with symptoms
Dissatisfaction	Dissatisfaction with one's diet
	Dissatisfaction with one's job
	Dissatisfaction with daily life subscale
